# XPRIZE HEALTHSPAN: A PATH TO ACCELERATE TRANSLATIONAL GEROSCIENCE

**DOI:** 10.1093/geroni/igae098.0321

**Published:** 2024-12-31

**Authors:** Jamie Justice

**Affiliations:** XPRIZE Foundation, Winston-Salem, North Carolina, United States

## Abstract

By targeting biological aging pathways with a single or combination of therapeutic treatments, it may be possible to restore function lost to age-related degradation of multiple organ systems. Interventions targeting aging pathways are being translated to humans, yet public perception, poor alignment and testing guidelines, and unclear regulatory and commercial pathways are barriers to clinical translation. XPRIZE Healthspan is a 7-yr $101M global competition to incentivize scientific teams to develop and test gerotherapeutics, accelerating progress from research & development to clinical trials. The winning team will demonstrate that their therapeutic treatment restores muscle, cognitive, and immune function in adults aged 50-80 years. The XPRIZE Healthspan concept was created through a deliberate process of scientific engagement and collaboration by 22 scientific advisors and technical experts across commercial and non-profit scientific sectors coupled with an open public comment period, and registration opened officially in July 2024. To date >420 investigative teams and companies from 53 countries have submitted an intent to compete. Teams span academic investigators, research networks or collaboratives, commercial start-ups, established biotech and pharmaceutical companies. A wide variety of potential therapeutics are proposed by teams for testing, including but not limited to AI-driven small molecule discovery, novel and repurposed pharmacologics, nutraceuticals, lifestyle interventions, devices, immunomodulatory agents, stem cell therapies, other biologics, and partial reprogramming – administered alone or in combination. In this talk we will review design of the global competition, describe frameworks for early-stage clinical trials, and provide an overview of the rapidly emerging field of translational geroscience.

